# Eco-friendly strategy for CO_2_ enrichment performance in commercial greenhouses based on the CO_2_ spatial distribution and photosynthesis

**DOI:** 10.1038/s41598-023-44200-9

**Published:** 2023-10-12

**Authors:** Yue Zhang, Daisuke Yasutake, Kota Hidaka, Kensuke Kimura, Takashi Okayasu, Masaharu Kitano, Tomoyoshi Hirota

**Affiliations:** 1 School of Agricultural Engineering, Jiangsu University, Jiangsu, China; 2 Faculty of Agriculture, Kyushu University, Fukuoka, 819-0395 Japan; 3 IoP Co-Creation Center, Kochi University, Nankoku, 783-8502 Japan; 4 NARO, Kyushu Okinawa Agricultural Research Center, Kurume, Fukuoka, 839-8503 Japan; 5 NARO, Institute of Agro-Environmental Sciences, Tsukuba, Ibaraki, 305-8604 Japan

**Keywords:** Energy science and technology, Fluid dynamics, Photosynthesis

## Abstract

CO_2_ enrichment is an essential environmental control technology due to its significantly enhancing effect on crop production capacity. Despite being a key energy consumer in protected agriculture (i.e. greenhouse systems), CO_2_ enrichment remains at a low energy use efficiency level, highlighting the need for developing more energy-efficiency strategies for CO_2_ enrichment. Therefore, this study employed the computational fluid dynamics (CFD) simulation method to replicate the CO_2_ diffusion process resulting from CO_2_ enrichment in three commercial strawberry greenhouses with varying geometric characteristics. Based on the CFD-simulated CO_2_ concentration distributions, the leaf photosynthetic rate was calculated using a mathematical model group. The CO_2_ enrichment efficiency was then analysed by calculating the ratio of increased photosynthesis across the cultivation area to the amount of energy (in CO_2_ equivalent) used. The efficiency peaked when the average CO_2_ concentration was approximately 500 μmol mol^−1^, thereby providing guidance for determining the target concentration of CO_2_ enrichment in production. Although this study is limited as the CFD simulation only considered a typical short-period CO_2_ enrichment event, future research will provide a broader analysis by considering changes throughout the day.

## Introduction

Energy utilisation and CO_2_ emissions are strongly interconnected with climate change and global warming, which pose significant threats to ecosystems. Agricultural activities account for ~ 30% of total global energy consumption^1^ and contribute to ~ 20% of total anthropogenic CO_2_ emissions^2^. Protected horticulture, i.e., crop production in greenhouses, is considered an intensive farming system in terms of production, topping the energy consumers in the agricultural sector^3^. Unlike open field production, greenhouse production could control environmental parameters, such as temperature, humidity, CO_2_ concentration, wind speed and solar radiation^4^, and this would significantly contribute to crop productivity^5^. However, controlling environmental parameters can incur enormous energy consumption and markedly increase production costs^6^. Therefore, conducting accurate analyses of the performance of different existing environmental control methods is crucial. Such analyses can provide valuable guidance for optimising environmental control strategies, resulting in improved efficiency and maximised production capacity in greenhouses. Ultimately, this optimisation can lead to the best possible economic and environmental benefits.

CO_2_ enrichment has been widely used in modern greenhouses as an effective method of increasing crop yield^7,8^. Numerous studies conducted thus far have demonstrated its effectiveness in promoting crop photosynthesis, growth, yield and quality^1,9,10^. Owing to its readily available raw materials, moderate cost and easy control, kerosene-generated CO_2_ has become the predominant source for CO_2_ enrichment in Japanese greenhouses.

Using exhaust gas as a CO_2_ source could directly relate to fossil fuel consumption, so the efficiency of CO_2_ enrichment serves as a key parameter when considering economic and eco-friendly production practices. Researchers have proposed various evaluation criteria for assessing CO_2_ enrichment efficiency. Sánchez-Guerrero et al.^11^ reported an 8% average efficiency by employing the content of C-assimilates in the shoot part produced through CO_2_ enrichment and comparing it to the amount of CO_2_ used. Kuroyanagi et al.^12^ reported a 45.5% average efficiency by calculating the proportion of crop-absorbed CO_2_ in a closed greenhouse to the total CO_2_ used. These findings indicate significant room for improving efficiency in order to realise decreased costs and CO_2_ emissions.

In order to uncover more efficient and eco-friendly CO_2_ enrichment strategies, the CO_2_ enrichment performance in commercial production requires clarification. Many factors are closely related to this performance, including spatial distribution of CO_2_ in different greenhouses, its impact on photosynthesis and CO_2_ utilisation efficiency. While environmental controls can optimise the microclimates within greenhouses, they often introduce spatiotemporal heterogeneity in environmental parameters^13^. For example, Zhang et al.^14^ analysed the CO_2_ concentration distribution inside a greenhouse using different enrichment strategies, revealing its inherent dependence on enrichment methods. The study concluded that coordinating such heterogeneity with the distribution of crops inside greenhouse spaces is an important factor for effective production. With this consideration, clarifying the CO_2_ spatial distribution is essential for accurately assessing CO_2_ enrichment performance. However, given the huge size of commercial greenhouses, obtaining an accurate measurement of CO_2_ distribution through actual means remains challenging. Computational fluid dynamics (CFD) could overcome the identified problems, as it provides an effective method for elucidating the distribution of microclimate parameters within greenhouses^15^. CFD has been extensively applied and validated in studies to predict the distribution of environmental parameters, including temperature^16,17^, humidity^18,19^ and wind speed^20^. The application of CFD technology in stimulating CO_2_ distribution within greenhouses has been reported. Roy et al.^21^ and Boulard et al.^22^ analysed CO_2_ distribution simulations in greenhouses under CO_2_ enrichment.

The primary objective of implementing CO_2_ enrichment is to enhance crop photosynthesis, thereby increasing greenhouse productivity on a larger scale^23^. This process is highly intricate, as it involves altering CO_2_ concentrations to influence crop photosynthesis through various physiological and biochemical reactions. Therefore, evaluations based only on environmental parameters are incomprehensive and fail to incorporate the ultimate target, photosynthesis. Photosynthesis-based evaluations of CO_2_ enrichment require a clearer understanding of the overall distributions of photosynthesis. Zhang et al.^24^ visualised leaf photosynthetic rate distribution inside a greenhouse by applying a combined plant–environment-coupled mathematical model group. In that research, environmental parameters inside the greenhouse were simulated using a CFD model, as it provides a convenient method for estimating photosynthesis distribution in large-scale cultivation facilities. Based on the data on photosynthetic capacity change, precise CO_2_ utilisation efficiency could be obtained by quantitatively analysing the relationship between the photosynthetic capacity change of the greenhouse and the amount of CO_2_ used.

Furthermore, the geometric features of a greenhouse (e.g. aspect ratio) could greatly influence microclimates inside greenhouses and energy consumption^25^. In commercial production, greenhouses often vary in aspect ratios and scales based on the available land owned by the cultivator. This variation introduces complex effects on environmental control. Therefore, examining the application of CO_2_ enrichment in different commercial greenhouses can provide a more objective and comprehensive evaluation, offering valuable insights for commercial production with greater guiding significance. While recent studies have explored new crop-localised enrichment methods^24,26,27^, burning fossil fuels and directly releasing the resulting CO_2_ into greenhouse air remains the most widely utilised method for CO_2_ enrichment. This is primarily due to the easy accessibility of raw materials, moderate cost and simplicity of control associated with this approach^28^. Therefore, performing an analysis of CO_2_ enrichment performance in actual commercial greenhouses with different geometric features under the direct release of fossil fuel-generated CO_2_ gas is crucial.

In this study, experiments were conducted in three commercial strawberry greenhouses with typical length and width, which are representative geometric features of commercial greenhouses in Japan, each greenhouse being equipped with a CO_2_ generator that produced CO_2_ gas by burning fuel (kerosene). The objectives of this study were to comprehensively analyse CO_2_ enrichment performance in commercial production and to propose suitable improvement measures. These aims were achieved following these steps: (1) developing unsteady 3D CFD models to simulate the CO_2_ diffusion process after enrichment in three different commercial greenhouses, (2) combining CFD with a photosynthetic model to simulate the leaf photosynthetic rate distribution on a greenhouse scale, and (3) evaluating the CO_2_ enrichment efficiency in the different greenhouses by considering their construction characteristics and discussing the appropriate enrichment strategies. Notably, the main novelty of the study is its visualisation of the distributions of CO_2_ and photosynthesis within greenhouses during CO_2_ enrichment and the establishment of their relationship with greenhouse geometric features.

## Material and methods

### Experimental greenhouses with CO_2_ enrichment

**Figure 1:**
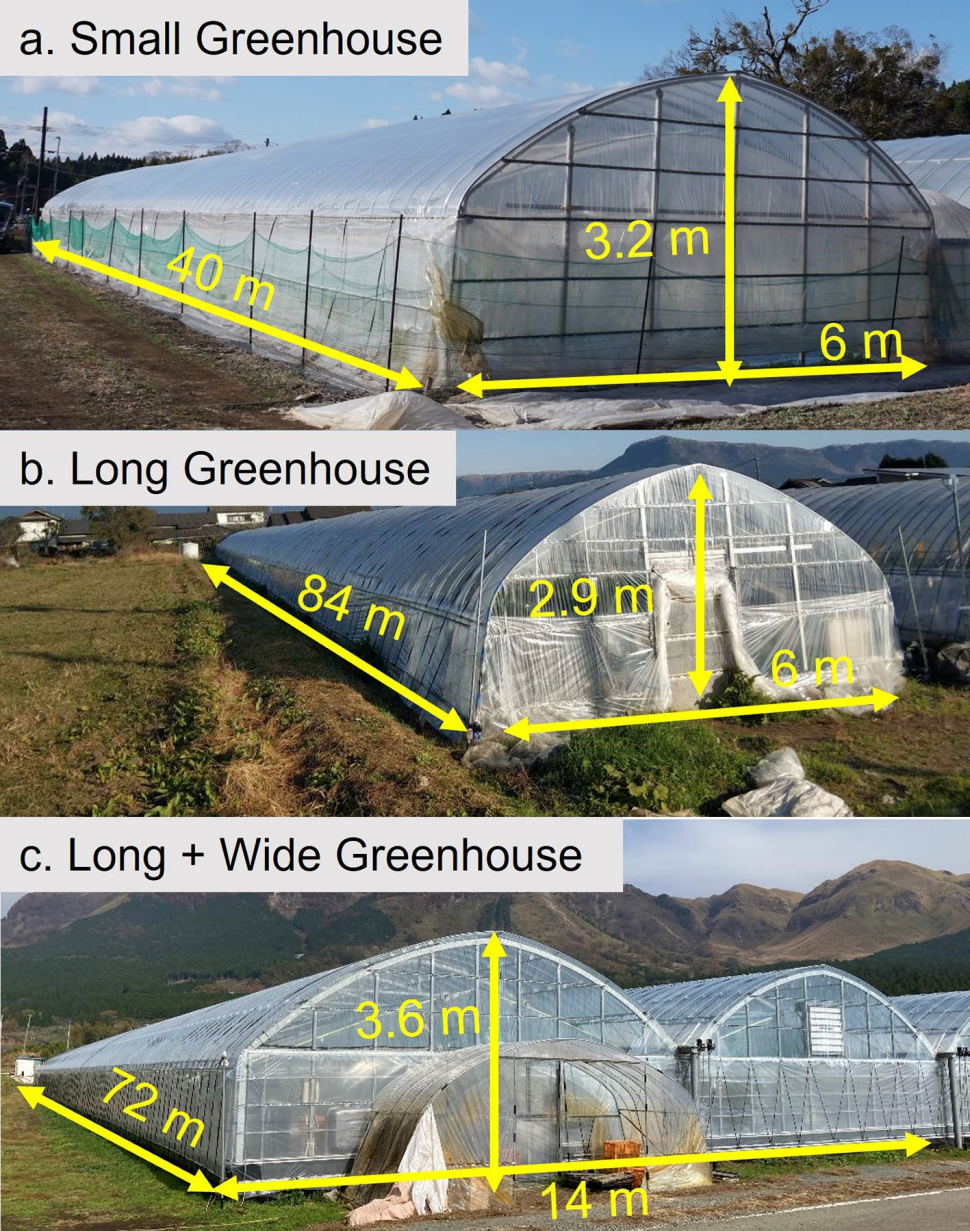
Photographs of the three greenhouses of Small Greenhouse (**a**), Long Greenhouse (**b**) and Long + Wide Greenhouse (**c**) used in this study.

To evaluate CO_2_ enrichment performance under commercial conditions, three different greenhouses located in Aso City, Kumamoto Prefecture, Japan (32° 57′ 31 N, 131° 03′ 20.9′′ E) were selected according to their sizes and shapes (Fig. 1): Small Greenhouse (length, 40 m; width, 6 m; height, 3.2 m), Long Greenhouse (length, 84 m; width, 6 m; height, 2.9 m) and Long + Wide Greenhouse (length, 72 m; width, 14 m; height, 3.6 m). Table 1 contains the specific geometric information for the three greenhouses. During the experiment, all greenhouses were managed under conditions of normal commercial production^27^ and equipped with a CO_2_ generator (ZO451, FULTA ELECTRIC MACHINERY CO., LTD., Japan), which generated CO_2_ gas by burning kerosene and released it directly into the space inside. In order to accurately control the CO_2_ enrichment process, a CO_2_ concentration monitoring device was employed in the experimental greenhouses. CO_2_ sensors were arranged near the strawberry at the greenhouse centre to monitor the CO_2_ concentration inside the greenhouse. In this way, the CO_2_ concentration changes could be observed in real-time while controlling it manually or automatically by the on–off action of the CO_2_ generator. In commercial production, the target of CO_2_ concentration was 1000 μmol mol^−1^, and the enrichment automatically stopped when the CO_2_ concentration reached the target value. According to the farmer’s experience and our in-site observation of the CO_2_ concentration, ~ 10 min was needed for the concentration to reach the target value, so 10 min was chosen as the enrichment duration. During CO_2_ enrichment, greenhouses were kept closed, and no other environmental control measures were applied. Strawberry plants (*Fragaria* × *ananassa* Duch.) were planted in plastic cultivation beds at a height of ~ 1.1 m. The plants were grown in a substrate with an approximate volume of 2 L per plant and irrigated using a nutrient solution.

**Table 1:** Geometric information for the three greenhouses.

Objects	Length (m)	Width (m)	Height (m)	Area (m^2^)	Volume (m^3^)
Small Greenhouse	40	6	3.2	240	640
Long Greenhouse	84	6	2.9	508	1080
Long + Wide Greenhouse	72	14	3.6	1008	2912

### Measurement and data collection

**Figure 2:**
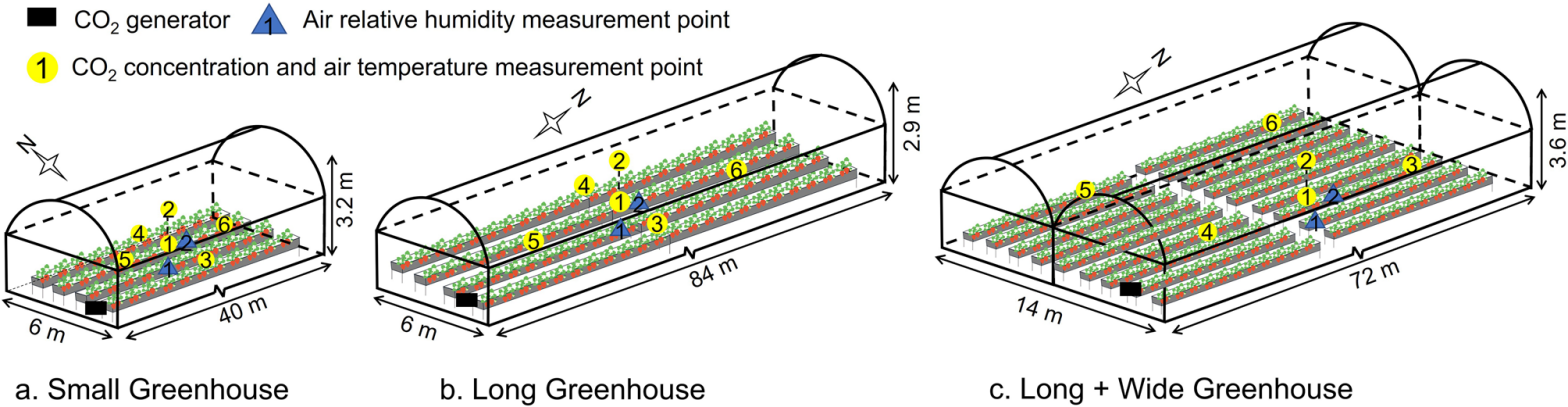
Dimensions of three greenhouses: Small Greenhouse (**a**), Long Greenhouse (**b**) and Long + Wide Greenhouse (**c**). The height for CO_2_ and air temperature measurement points 1, 3, 4, 5 and 6 was 1.5 m and Point 2 was 2.5 m. The height of the air relative humidity measurement Point 1 was around the canopy, and Point 2 was 1.5 m.

**Figure 3:**
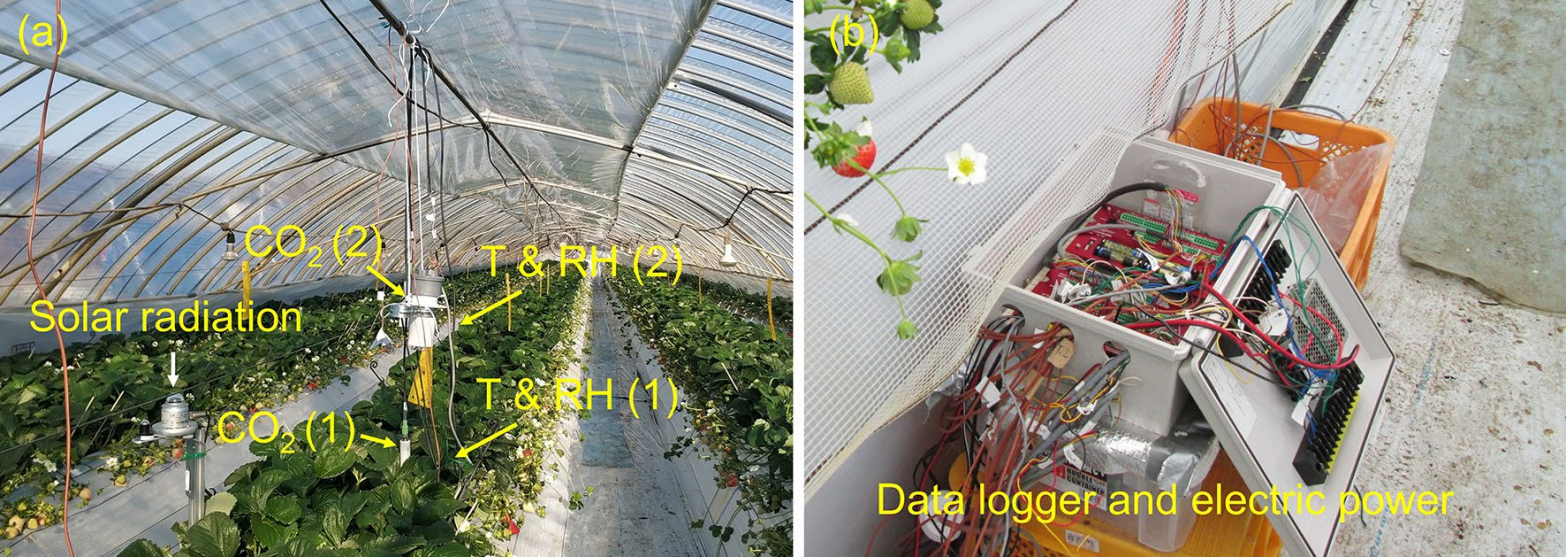
Photographs of the installation of the sensors at the Long Greenhouse centre (**a**) and the data acquisition system (**b**). The sensors included solar radiation sensors, CO_2_ sensors (No. 1 and 2) and temperature and relative humidity (T & RH) sensors (No. 1 and 2).

During the experiment, various environmental parameters (temperature, humidity, solar radiation, CO_2_ concentration, wind speed) within the greenhouses were measured to provide the data required for setting up the simulation environment and verifying model accuracy. These measurements were made in December 2019 and December 2020. Upon multiple repetitions, four replicates were made for the Small and Long Greenhouses and two replicates were made for the Long + Wide greenhouse, enabling model validation (for air temperature, air relative humidity and CO_2_ concentration, see Section "CFD simulation accuracy validation"). The experimental greenhouse was kept unventilated during the measurements. CO_2_ concentrations were measured using CO_2_ sensors (GMP 252, Vaisala, Finland; an accuracy of ± 40 μmol mol^−1^ and a measurement range of 0–2000 μmol mol^−1^) at six points inside each greenhouse. The location and number of sensors were determined by referring to previous studies on CFD analysis^21,22,29^. The air temperature was measured at the same position for CO_2_ measurement using T-type thermocouples. The thermocouples were covered with aluminium shield which enables sun protection and natural ventilation. Figures 2 and 3 show the locations of each CO_2_ and air temperature measurement point, respectively. The height of measurement points (1, 3, 4, 5 and 6) was 1.5 m, and Point 2 was 2.5 m. This arrangement enabled a more comprehensive grasp of the CO_2_ and air temperature distribution at various locations inside the greenhouses. Solar radiation was measured using three pyranometers (PCM01-SD, PREDE, Japan), where two were located at the greenhouse centre and one was located near the greenhouse roof. The relative humidity of greenhouse air was measured using two humidity sensors (HMP60, Vaisala, Finland; an accuracy of ± 3% and a measurement range of 0%–100%), and these sensors were located around the greenhouse centre, where one was at a canopy height and the other at a 1.5 m height. The generator outlet wind speed was measured using a thermal anemometer (VA21, IET, Japan; an accuracy of ± 3% and a measurement range of 0–40 m s^−1^). The above data were recorded using a data logger (MIJ-01, Environmental Measurement Japan) at a 10-s interval. Temperatures at various locations (soil, PO plastic film and cultivation bed) in the greenhouse were measured using a mobile type of temperature sensor (Tr-52i, T&D, Japan; an accuracy of ± 0.3 °C and a measurement range of − 60–155 °C). Before starting the experiment, all sensors were placed under the same standard environmental conditions and corrected for differences in each instrument.

### CFD simulation

#### Basic equations

Commercial software (FLUENT, ANSYS) was used to simulate the microclimate and CO_2_ diffusion processes inside the greenhouse. Due to its sealed nature, the airflow within the greenhouse was primarily driven by natural convection, with the temperature difference between different locations serving as the main influencing factor. To verify the diffusion process of CO_2_ gas inside the greenhouse, unsteady simulations were used in this study. The ideal gas law was chosen to simulate air density change caused by the temperature difference, after which the actual measured temperatures and radiations of various boundaries were used to create a realistic temperature and natural convection environment. The governing equation for physical quantity *φ* (mass, energy and monument) can be written as follows^30^:

1 $$\frac{\partial \varphi }{\partial t} + \frac{\partial \varphi }{\partial {x}_{j}}({u}_{j}\varphi ) = \frac{\partial }{\partial {x}_{j}}({\Gamma }_{\varphi }\frac{\partial \varphi }{\partial {x}_{j}}\text{)} + {S}_{\varphi }$$

where *x*_*j*_ and *u*_*j*_ represent the coordinate (m) and velocity component (m s^−1^) in the *j*th direction, *Γ*_*φ*_ pertains to the diffusion coefficient of *φ*, and *S*_*φ*_ is the source term of *φ.*

The airflow inside the greenhouse was simulated using the standard *k–ε* model because it showed good accuracy in many dispersion simulations and greenhouse studies^15,16,31–35^. The discrete ordinates (DO) model was used to calculate the solar radiation inside the greenhouse, and the canopy was considered a semi-transparent medium with an absorption coefficient of 0.46 and a refractive index of 2.77^36^.

#### Crop models

The structure of a canopy can affect airflow, inducing a blocking effect. To simulate this effect, the canopy was used as a porous medium—the most common method for simulating crop canopy in CFD studies^15,37^. Using this method, the canopy-induced resistance could be converted into a sink term in the momentum equation, written as follows:

2 $${S}_{\mathrm{m}} = -\rho \cdot LAD\cdot D\cdot {v}^{2}$$

where $\rho$ (kg m^−3^) is air density, *LAD* (m^2^ m^−3^) pertains to leaf area density defined as the leaf area index divided by the canopy height, *v* (m s^−1^) refers to the wind speed and *D* represents the drag coefficient. Notably, the constant 0.32 was adopted for *D* in this study because it has been applied in many simulation studies on greenhouse environments and showed good accuracy for different crops^17,22,30,37,38^.

The strawberry leaf transpiration can cause mass–energy exchange between the crop canopy and greenhouse environment, thereby altering environmental parameters such as humidity and temperature around the canopy. To simulate these processes, the leaf transpiration rate was calculated using plant physioecological process-based models^39^, which were also used to calculate canopy-generated water vapour, which was added to the simulation as a source term:

3 $$T\mathrm{r} = {\left(\left({{g}_{aw}}^{-1}\right) + \left({{g}_{sw}}^{-1}\right)\right)}^{-1}\left(VPD/{P}_{a}\right)$$

4 $${S}_{w} = T\mathrm{r}\cdot LAD$$

where *T*r (kg m^−2^ s^−1^) is the leaf transpiration rate, ${g}_{aw}$ (mol m^−2^ s^−1^) is leaf-boundary-layer conductance to H_2_O, ${g}_{sw}$ (mol m^−2^ s^−1^) is stomatal conductance to H_2_O, *VPD* (kPa) is leaf-to-air vapour pressure deficit and* P*_a_ (101.3 kPa) is atmospheric pressure.

The heat absorbed by transpiration can be written in the following form and added to the simulation as a sink term of energy:

5 $${S}_{h} = {H\cdot T}\mathrm{r}\cdot LAD$$

where *H* (J kg^−1^) is the heat of vapourisation.

#### Settings for CFD simulation

An ICEM-created unstructured mesh was used in this study, and 0.8, 1.2 and 1.5 million elements existed for Small, Long and Long + Wide greenhouses, respectively. The minimum orthogonal quality values for Small, Long and Long + Wide greenhouses were 0.3, 0.23 and 0.28; the maximum aspect ratios were 12.6, 14.9 and 13.4; skewness values were 0.69, 0.76 and 0.71, respectively.

In the simulation, temperature data obtained from actual measurements were used for all the parts inside the greenhouse. The air outlet of the CO_2_ generator was set as a mass flow inlet, and the airflow rate was obtained as follows:

6 $$W = {\rho }_{a}\cdot {v}_{a}\cdot {A}_{o}$$

where *W* (kg s^−1^) is the mass flow rate, *ρ*_a_ (kg m^−3^) is air density, *A*_o_ (m^2^) is the generator outlet area and *v*_a_ (m s^−1^) is the airflow velocity at the generator outlet.

Table 2 contains the specific settings and values for each boundary inside the greenhouse. Table 3 presents the physical parameters of the various materials used in the simulation. The average solar radiation amounts received by the canopy during the measurement (10 min) were 365, 178 and 204 W m^−2^ in Small, Long and Long + Wide greenhouses, respectively.

**Table 2:** Specific settings for each boundary in Small, Long and Long + Wide greenhouses.

Location	Kinematic conditions	Temperature conditions	Solar radiation	Mass conditions
Walls	No slip wall	Small: 12.2 °C, Long: 12 °C, Long + Wide: 12.3 °C	Semi-transparent	Null flux
Ground	No slip wall	Small: 17 °C, Long: 15.8 °C, Long + Wide: 16.5 °C	Opaque	Null flux
Cultivation bed	No slip wall	Small: 16.9 °C, Long: 14.7 °C, Long + Wide: 18.8 °C	Opaque	Null flux
Generator outlet	Mass flow inlet	Small: 65 °C, Long: 70 °C, Long + Wide: 70 °C	–	Small: 0.64 kg s^−1^ (Air) and 2.9 g s^−1^ (CO_2_), Long: 0.63 kg s^−1^(Air) and 2.86 g s^−1^ (CO_2_), Long + Wide: 0.63 kg s^−1^(Air) and 2.9 g s^−1^ (CO_2_)

**Table 3:** Specific parameters for the materials used in the simulation.

Objects	Density (kg m^−3^)	Specific heat (J kg^−3^ °C^−1^)	Thermal conductivity (W m^−1^ °C^−1^)	Absorption coefficient	Reflective coefficient	Refractive index
Air	Incompressible-ideal-gas	Mixing-law	0.024	0.15	0.00	1.00
Wall	900	2550	0.29	0.10	0.10	1.92
Soil	1600	2200	0.80	0.88	0.12	1.70

The SIMPLEC method and second-order upwind discretisation were used in simulations. The convergence criteria of residuals were automatically set by the software. The time step was set to 1 s to balance the calculation speed and accuracy.

### Photosynthesis analysis

#### Model description

The photosynthetic rate of a single leaf (*P)* was simulated using plant–environment-combined mathematical models^13^. The model group comprised biochemical photosynthesis^40^, stomatal conductance^41^, and single-leaf transport models for CO_2_ and heat. Table 4 contains the specific values of the main parameters used in the model. For the photosynthetic rate calculation, *P* was detained by the hyperbolic minimum of the Rubisco-limited rate (*P*_c_) and RuBP-limited rate (*P*_j_).

7 $${\theta }_{\mathrm{A}}{P}^{2} - P\left({P}_{\mathrm{c}}+{P}_{\mathrm{j}}\right) + {P}_{\mathrm{c}}{P}_{\mathrm{j}} = 0$$

8 $${P}_{\mathrm{c}} = \frac{{V}_{\mathrm{cmax}}\left({C}_{\mathrm{i}} - {\Gamma }^{*}\right)}{{C}_{\mathrm{i}} + {K}_{\mathrm{c}}\left(1 + \frac{O}{{K}_{\mathrm{o}}}\right)} - {R}_{\mathrm{d}}$$

9 $${P}_{\mathrm{j}} = \frac{J\left({C}_{\mathrm{i}} - {\Gamma }^{*}\right)}{4{C}_{\mathrm{i}} + 8{\Gamma }^{*}} - {R}_{\mathrm{d}}$$

where *θ*_A_ (0.99) represents the curvature in the transition from one limitation to the other, *V*_cmax_ (μmol m^−2^ s^−1^) represents the maximum rates of carboxylation, *C*_*i*_ (μmol mol^−1^) represents the leaf intercellular CO_2_ concentration, Γ* (μmol mol^−1^) represents the CO_2_ compensation point with no respiration, *J* (μmol m^−2^ s^−1^) represents the rate of electron transport dependent on light intensity, *K*_c_ (μmol mol^−1^) and *K*_o_ (μmol mol^−1^) represent the Michaelis constants for carboxylation and oxygenation (mmol mol^−1^), respectively, and *O* (mmol mol^−1^) represents the O_2_ concentration.

10 $$J = \frac{\phi I + {J}_{max} - {\{{(\phi I + {J}_{max})}^{2} - 4\phi I{\theta }_{J}{J}_{max}\}}^{0.5}}{2{\theta }_{J}}$$

where *I* (μmol m^−2^ s^−1^) represents the photosynthetic photon flux density, *J*_max_ (μmol m^−2^ s^−1^) represents the light-saturated rate of electron transport, *ϕ* (mol mol^−1^) represents the initial slope of the curve corresponding to the apparent quantum yield of the electron transport at low light conditions and *θ*_J_ (0.85) represents the curve convexity.

**Table 4:** Specific values of physiological parameters used for leaf photosynthetic rate calculation in this study^13^.

Parameters	Values	Units
*V*_cmax_	77.94	Μmol m^−2^ s^−1^
Γ*	42.75	Μmol mol^−1^
*J*_max_	160.91	Μmol m^−2^ s^−1^
*K*_c_	42.75	Μmol mol^−1^
*K*_o_	278.4	Mmol mol^−1^
*ϕ*	0.24	Mol mol^−1^
*θ*_J_	0.85	–
*O*	210	Mol mol^−1^
*g*_o_	0.012	Mol m^−2^ s^−1^
*g*_1_	3.21	–
*d*_leaf_	0.064	m

According to the gas diffusion theory of Fick’s law, the relationship between leaf photosynthetic rate and CO_2_ diffusion for a single leaf could be written as follows:

11 $$P = {g}_{ac}\left({C}_{a} - {C}_{s}\right) = {g}_{sc}\left({C}_{s} - {C}_{i}\right) = {(g}_{ac}^{-1} + {g}_{sc}^{-1}){)}^{-1}\left({C}_{a} - {C}_{i}\right)$$

where ${g}_{ac}$ represents the conductance of leaf-boundary-layer for CO_2_ transport, ${g}_{sc}$ represents the stomatal conductance for CO_2_, and *C*_a_ (μmol mol^−1^) and *C*_s_ (μmol mol^−1^) represent the CO_2_ concentration at the ambient air and leaf surface.

Following Medlyn et al.^41^, stomatal conductance for vapour (${g}_{sw}$) (mol m^−2^ s^−1^) could be calculated by:

12 $${g}_{sw} = {g}_{0} + 1.6(1 + \frac{{g}_{1}}{\sqrt{VPD}})\frac{P}{{C}_{s}}$$

where ${g}_{0}$ (mol m^−2^ s^−1^) represents the residual conductance and ${g}_{1}$ (3.21) represents the empirical constant. *VPD* (kPa) represents the leaf-air vapour pressure deficit.

The leaf-boundary-layer conductance (${g}_{ah}$) was calculated by the following equation:

13 $${g}_{ah} = 0.21(\frac{u}{{d}_{leaf}}{)}^{0.5}$$

where *u* represents wind speed and *d*_leaf_ (0.064 m) represents the strawberry leave characteristic dimension. ${g}_{ah}$ can be expressed as ${g}_{ac}$ and ${g}_{aw}$ using ${g}_{ac}$= ${g}_{aw}$/1.37 and ${g}_{aw}$ = 1.08 ${g}_{ah}$.

In this study, the effect of CO_2_ enrichment on greenhouse photosynthetic capacity could be demonstrated by using the CFD-simulated CO_2_ concentration values to calculate the leaf photosynthetic rates. The model accuracy has been validated in a previous study (Kimura et al., 2020).

#### Spatial analysis of leaf photosynthetic rate

**Figure 4:**
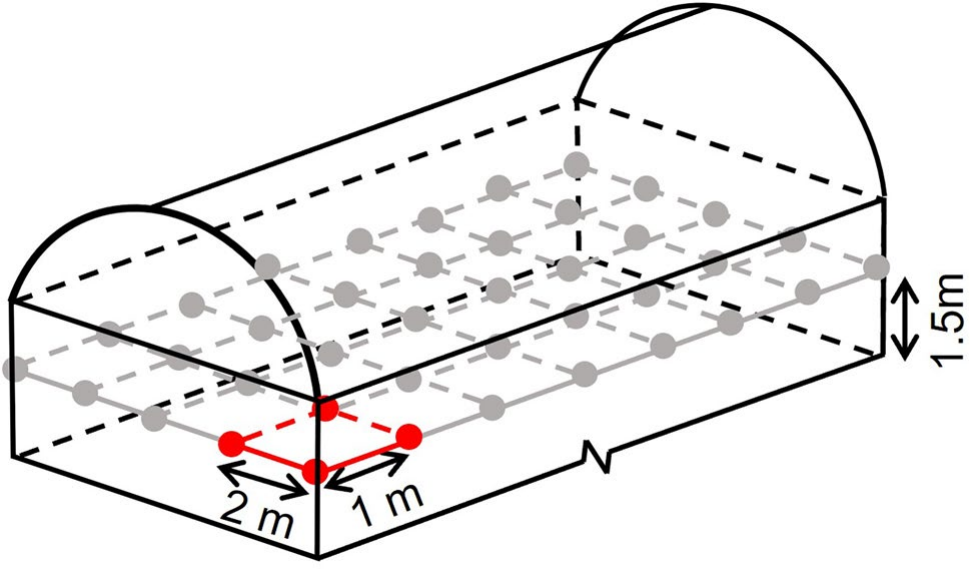
Schematic diagram of sampling points for photosynthetic rate in the greenhouse. The height of the sampling points was 1.5 m, and each point was separated by 2 m in the width direction and 1 m in the length direction.

To quantitatively analyse the spatial distribution of the photosynthetic rate at the plane of 1.5 m height, which was close to the strawberry canopy inside each greenhouse, evenly distributed sampling points in each greenhouse were selected, separated by 2 m in the width direction and 1 m in the length direction (Fig. 4).

Due to the different areas of each greenhouse, 123, 255 and 511 sampling points were observed in Small Greenhouse, Long Greenhouse and Long + Wide Greenhouse, respectively.

#### Efficiency of CO_2_ enrichment

In order to quantitatively analyse the impact of CO_2_ enrichment on greenhouse overall photosynthetic capacity and energy utilisation efficiency, the efficiency of CO_2_ enrichment (ECE, mmol mol^−1^) was calculated. ECE was defined as the ratio of the increase in greenhouse photosynthetic capacity to the amount of CO_2_ used; the equation can be written as follows:

14 $$\mathrm{ECE }= \frac{\left({P}_{a} - {P}_{400}\right){A}_{g}}{M}$$

where* P*_a_ (mmol m^−2^ s^−1^) is the average leaf photosynthetic rates in the 1.5 m height plane, *P*_400_ (mmol m^−2^ s^−1^) is the leaf photosynthetic rate when the air CO_2_ concentration is 400 μmol mol^−1^, *A*_g_ (m^2^) is the greenhouse ground area and *M* (mol s^−1^) is the amount of energy consumption (equivalent to CO_2_).

Changes in ECE according to different levels of CO_2_ usage in the respective greenhouses were calculated. The enrichment effects with different levels of CO_2_ usage were simulated by changing the mass flow rate from the CO_2_ generator (from 0.05 to 0.5 kg s^−1^). After that, the average CO_2_ concentration in the 1.5 m height plane and the corresponding ECE were analysed for different levels of CO_2_ usage.

### Ethical statement

The plant collection and use were in accordance with all the relevant guidelines.

## Results and discussion

### CFD simulation accuracy validation

**Figure 5:**
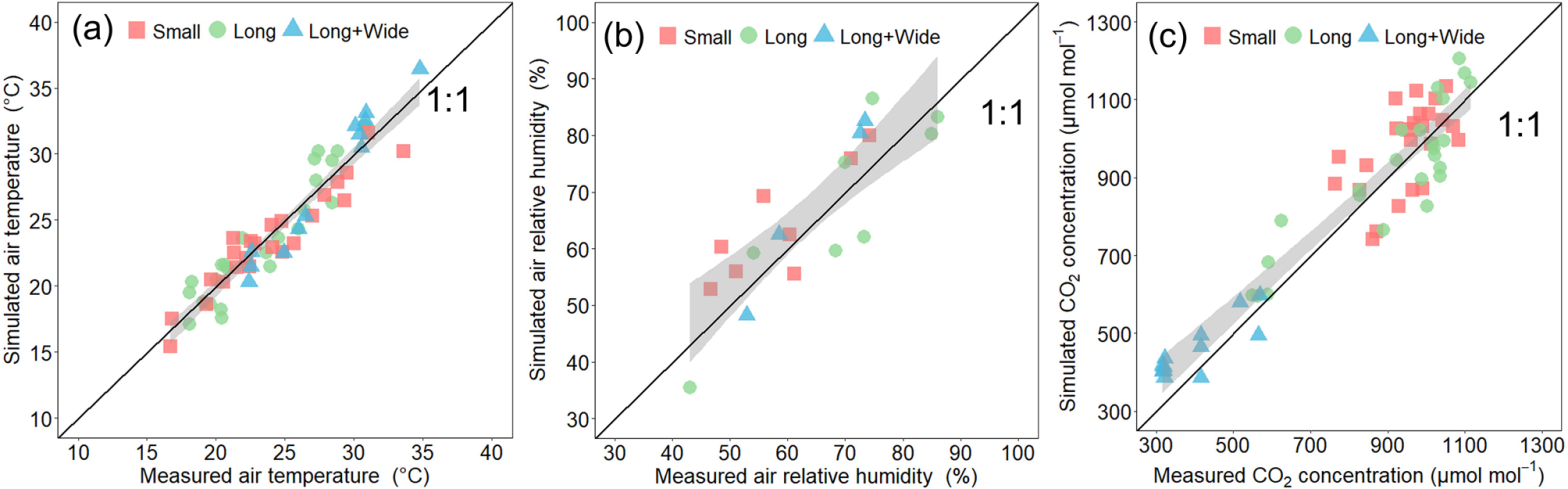
Relationship between measured and simulated values for air temperature (**a**), air relative humidity (**b**) and air CO_2_ concentration (**c**) in the three greenhouses (Small, Long and Long + Wide Greenhouses). The shaded area indicates the 95% confidence interval.

Figure 5 shows the comparison of measured and CFD-simulated values of air temperature, relative humidity and CO_2_ concentration at corresponding measurement points for three greenhouses. The mean absolute errors of air temperature, relative humidity and CO_2_ concentrations were 1.3 °C, 6.9% and 79.2 μmol mol^−1^, indicating that the CFD simulations achieved good accuracy. Considering the accuracy obtained in previous studies^21,22,29^, the error values were within the normal range for CO_2_ simulation using CFD. These results demonstrate that the CFD model accurately simulated the diffusion process of CO_2_ in different greenhouses. In addition, the simulation results maintained good accuracy with different greenhouses under varying environmental conditions, indicating that the CFD model had good adaptability.

### CO_2_ spatial distribution in the three greenhouses

**Figure 6:**
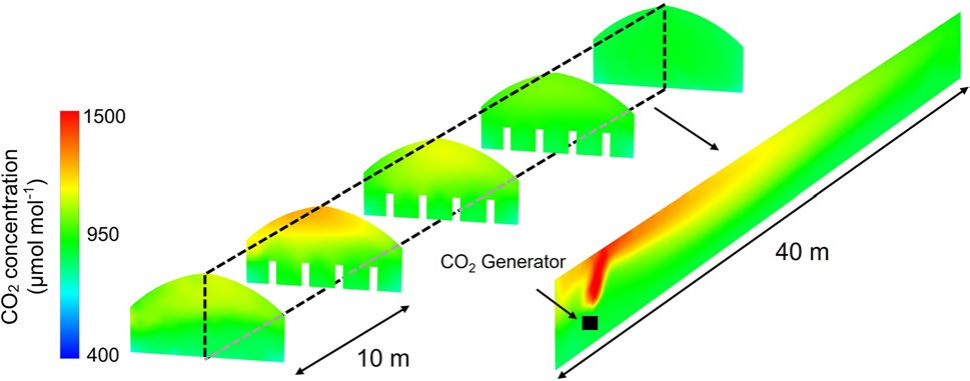
CO_2_ concentration distribution inside the Small Greenhouse after 10 min enrichment.

Figure 6 demonstrates the CO_2_ concentration distribution inside the Small Greenhouse after 10 min enrichment. A significant concentration difference in the height direction of the greenhouse could be seen, which was caused by the upward convection of CO_2_ gas. Because CO_2_ was produced by combustion, it had a much higher temperature (65 °C) than the greenhouse air (27 °C), thereby causing CO_2_ gas to drift to the greenhouse’s upper part and gather there. Therefore, this could limit the effects of CO_2_ enrichment since the crops are located in the lower regions. However, the difference in CO_2_ concentration along the length direction was not so large due to its smaller size; the concentration at each location was maintained at ~ 1000 μmol mol^−1^. This had positive significance for maintaining a uniform CO_2_ concentration environment in the cultivation area.

**Figure 7:**
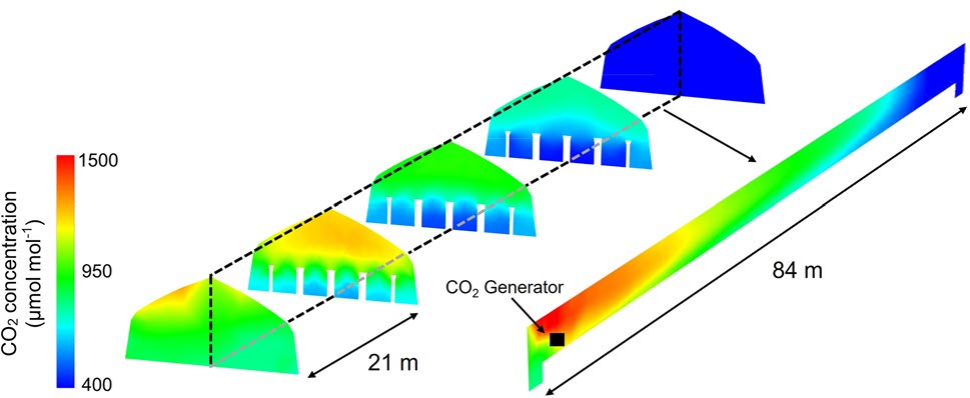
CO_2_ concentration distribution inside the Long Greenhouse after 10 min enrichment.

Figure 7 demonstrates the CO_2_ concentration distribution inside the Long Greenhouse after 10 min enrichment. Given that this greenhouse used the same method to produce CO_2_, the same problem also occurred in the height direction near the CO_2_ generator. However, unlike the Small Greenhouse, a significant CO_2_ concentration gradient was observed along the length direction mainly due to the extremely long length (> 80 m) of the greenhouse. In the region near the generator, the CO_2_ concentration could reach ~ 1500 μmol mol^−1^, while in the region far from the generator, the CO_2_ concentration did not significantly increase. The uniformity of CO_2_ concentration distribution should directly influence the effect of CO_2_ enrichment^7^. Such uneven distribution could reduce the overall effect of increasing yield, posing difficulties in applying a reasonable control strategy of CO_2_ enrichment. In many modern greenhouses, environmental control devices are often regulated by real-time monitoring of environmental parameters, usually measured at one point (e.g. around greenhouse centre)^13^. For CO_2_ enrichment, setting relevant ranges to control the usage of CO_2_ generators is necessary, but uneven distribution makes it difficult to select the sampling points and target concentrations in greenhouses with longer lengths.

**Figure 8:**
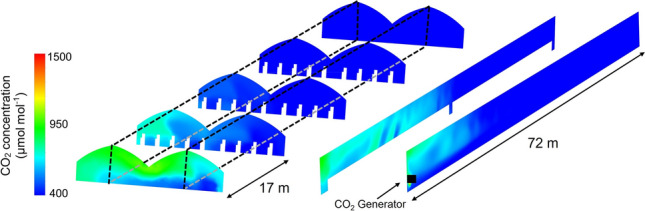
CO_2_ concentration distribution inside the Long + Wide Greenhouse after 10 min enrichment.

Figure 8 demonstrates the CO_2_ concentration distribution inside the Long + Wide Greenhouse after 10 min enrichment. The application of CO_2_ enrichment in this greenhouse produced quite different results from those in the previous two greenhouses. Notably, the CO_2_ concentration in most areas of the greenhouse did not increase significantly, mainly due to the huge volume of the greenhouse, being 4 and 2.5 times the volume of the Small and Long Greenhouses, respectively. Thus, the current application of CO_2_ for greenhouses with a large volume significantly failed to achieve good performance.

**Figure 9:**
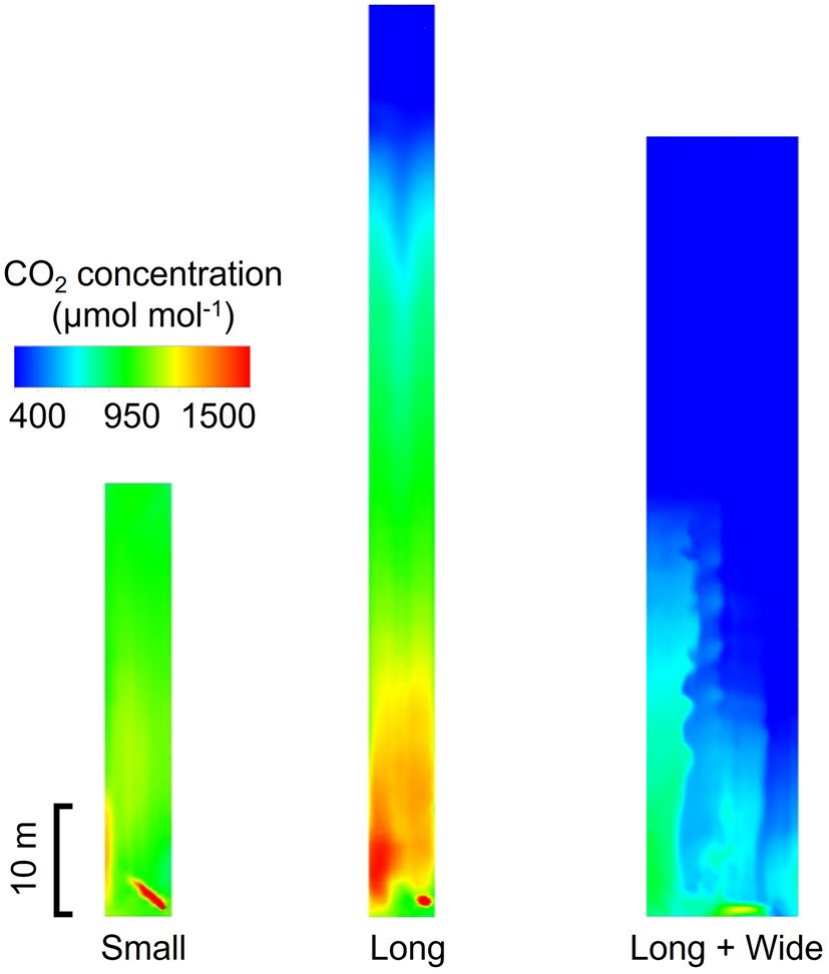
CO_2_ concentration distribution inside the 1.5 m height plane after 10 min enrichment in three greenhouses (Small, Long and Long + Wide Greenhouses).

**Figure 10:**
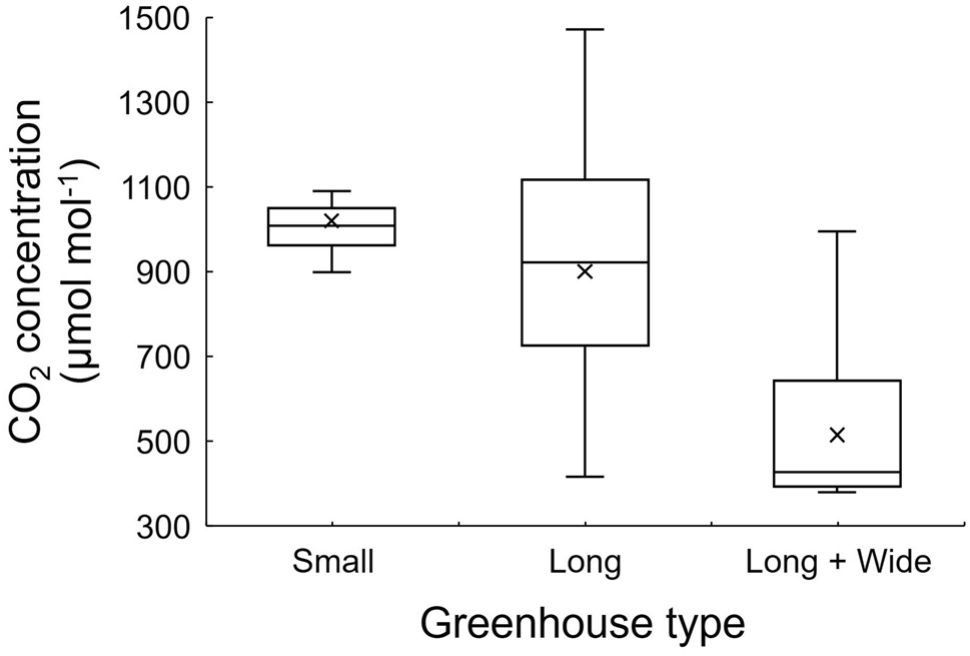
Box plot for CO_2_ concentration at the 1.5 m height plane 10 min after enrichment for three greenhouses (Small, Long and Long + Wide Greenhouses).

Because the strawberry canopy CO_2_ concentration is considered the most important factor when evaluating the effect of enrichment, the spatial distribution of CO_2_ concentration at the 1.5 m height plane was also analysed (Fig. 9). Figure 10 shows a statistical analysis of the CO_2_ concentration within the plane, revealing that the CO_2_ enrichment performance in the three greenhouses differed significantly. In the Small Greenhouse, the difference in CO_2_ concentration in the cultivation area was small, and the overall concentration was maintained at 900–1100 μmol mol^−1^. Given the absence of extremely high or low concentrations, the current measures of CO_2_ enrichment in this greenhouse achieved a relatively ideal state. In the Long Greenhouse, although the median and average values of CO_2_ concentration were both maintained at ~ 900 μmol mol^−1^, a relatively ideal state, obvious extreme values in the highest and lowest CO_2_ concentrations were observed. With the highest concentration reaching close to 1500 μmol mol^−1^, the lowest value stayed at 400 μmol mol^−1^ level. Notably, most of the recommended target CO_2_ concentration from the literature is between 600 and 1000 μmol mol^−1^ ^8,10,42,43^, and excessive CO_2_ has a limited effect on promoting photosynthesis, indicating that the state for this greenhouse would reduce the efficiency of CO_2_ enrichment. Therefore, the primary improvement requirement for this greenhouse type is to achieve a more uniform distribution of CO_2_ concentration with the interior. In the Long + Wide Greenhouse, the problem was mainly that the effect of CO_2_ enrichment was not noticeable. Although some areas had a CO_2_ concentration of 1000 μmol mol^−1^ near the CO_2_ generator, the overall CO_2_ concentration level of the cultivation area remained around 400 μmol mol^−1^. Therefore, the primary improvement should be increasing CO_2_ concentration in the greenhouse.

### Analysis of leaf photosynthetic rate in three greenhouses

**Figure 11:**
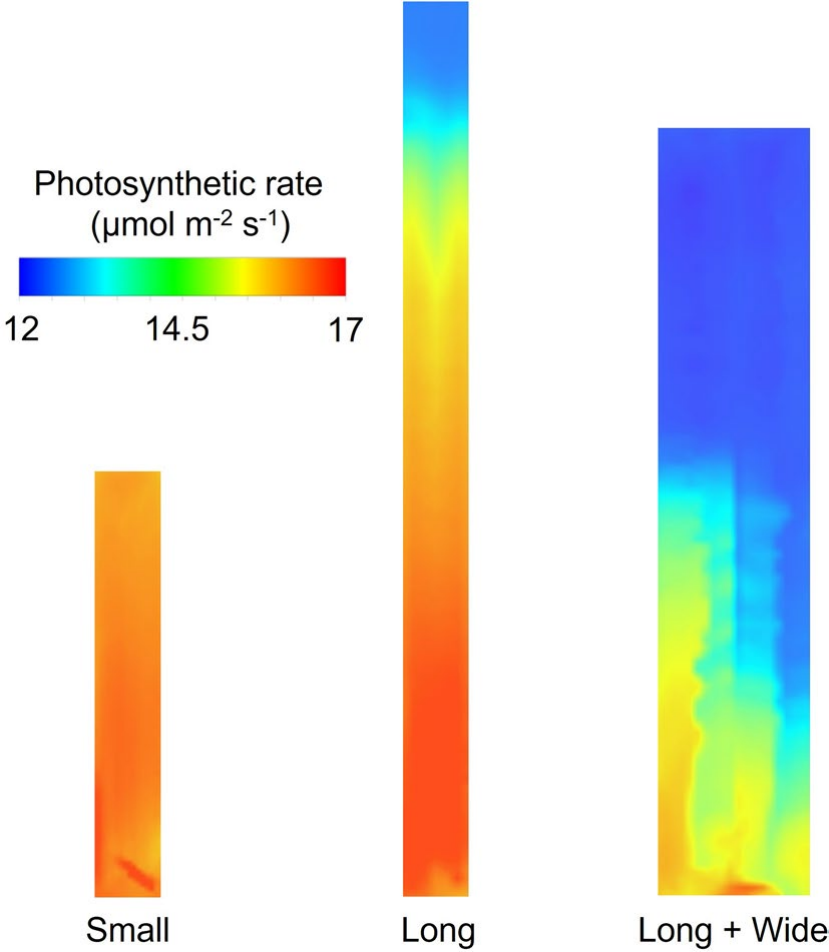
Distribution of the leaf photosynthetic rate at the 1.5 m height plane after 10 min enrichment in three greenhouses (Small, Long and Long + Wide Greenhouses).

**Figure 12:**
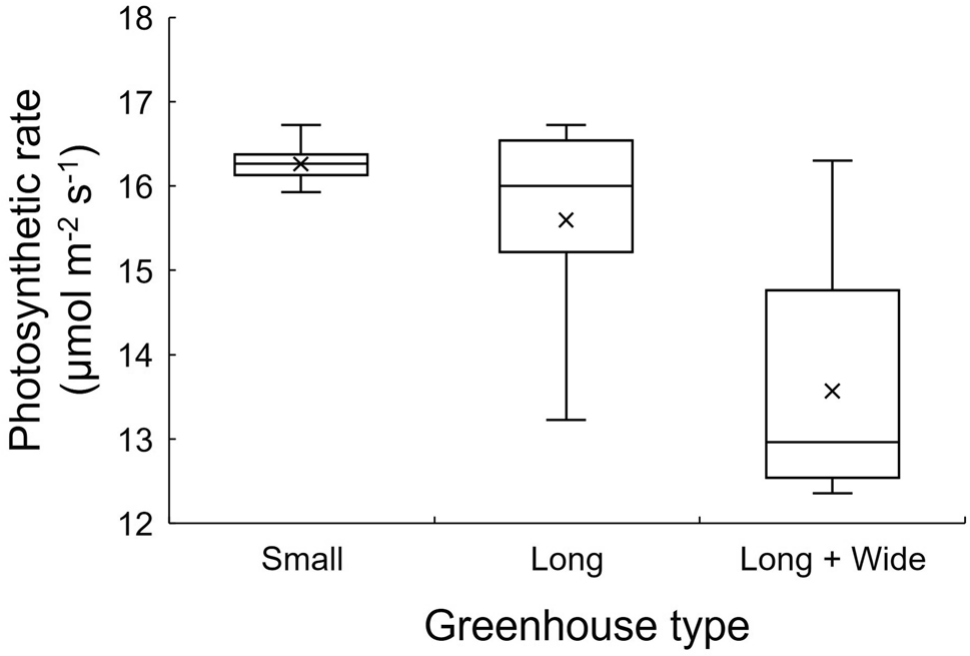
Box plot for leaf photosynthetic rate at the 1.5 m height plane 10 min after enrichment for three greenhouses (Small, Long and Long + Wide Greenhouse).

The biochemical process of leaf photosynthesis should be considered when conducting CO_2_ enrichment within greenhouses^7,13^. Figures 11 and 12 illustrate the spatial distribution and statistics of the leaf photosynthetic rate at the 1.5 m height plane for the three greenhouses, respectively, intuitively revealing the effect of increasing CO_2_ concentration on crop photosynthesis. Given the constancy assumption of environmental parameters other than the CO_2_ concentration, the distribution of leaf photosynthetic rate was attributed to the CO_2_ concentration distribution. The photosynthetic rate was unevenly distributed in the Long and Long + Wide Greenhouses compared to the Small Greenhouse, possibly explaining why an insignificant increasing effect of CO_2_ enrichment was observed in those greenhouses. Considering the overall photosynthetic rate variations in the greenhouses, the average values for the three greenhouses increased by 28.7%, 23.4% and 7.4%, respectively, for the Small, Long and Long + Wide Greenhouses, compared to their status under the condition of CO_2_ concentration at 400 μmol mol^−1^. As shown in Fig. 12, the difference in the photosynthetic rate at respective locations in the Small Greenhouse was extremely small, as most areas had photosynthetic rates staying above the 16 μmol m^−2^ s^−1^ level. In the Long Greenhouse, the maximum value of the photosynthetic rate changed slightly compared to the Small Greenhouse, which can be attributed to the nonlinear relationships between the photosynthetic rate and CO_2_ concentration, although an obvious variation existed in the uniformity of the photosynthesis rate. In addition, only half of the area had a photosynthetic rate exceeding 16 μmol m^−2^ s^−1^, and the lowest photosynthetic rate reached a level close to 13 μmol m^−2^ s^−1^. In the Large + Wide Greenhouse, the uneven distribution of photosynthetic rate still existed, and the effect of increasing this rate significantly dropped to 12.5 μmol m^−2^ s^−1^. More than half of the area had photosynthetic rates below 13 μmol m^−2^ s^−1^.

### ECE analysis in three greenhouses

**Figure 13:**
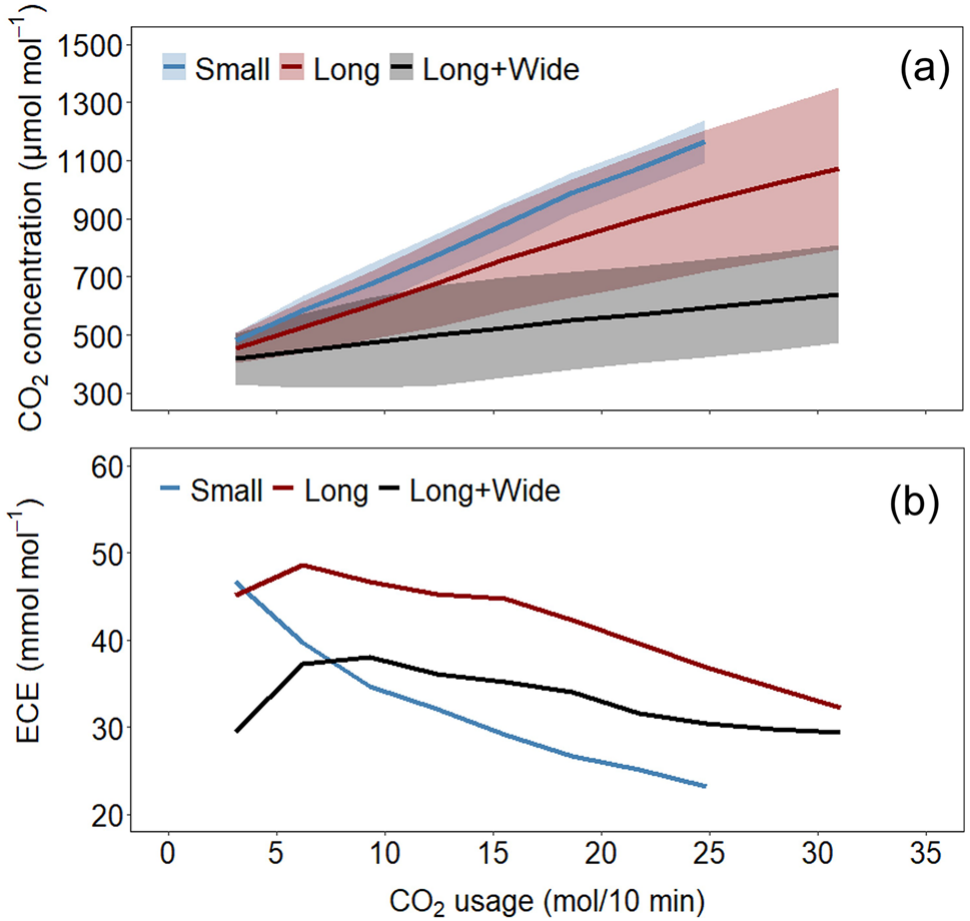
The relationships of the average CO_2_ concentration at the 1.5 m height plane with standard deviation shown as the colour band (**a**) and the efficiency of CO_2_ enrichment (ECE) (**b**) with the amount of CO_2_ usage in three greenhouses (Small, Long and Long + Wide Greenhouses).

Figure 13 demonstrates the change in average CO_2_ concentration in the 1.5 m height plane and CO_2_ use efficiency under different amounts of pure CO_2_ gas (mol) enriched in the greenhouse. Although most researchers have provided an estimated range of suitable CO_2_ concentrations 600–1000 μmol mol^−1^ ^8,10,42,43^, clarifying the changes in greenhouse photosynthetic capacity and energy use efficiency under different CO_2_ usage conditions is needed for improved performance and efficiency. The average CO_2_ concentration over the greenhouse plane exhibited an obvious linear relationship with CO_2_ usage (Fig. 13). The resulting slope gradually became smaller as the volume increased because the greenhouses had volume variations. Another important factor affecting the concentration change is that the inhomogeneity (standard deviation) of CO_2_ concentration in the cultivation planes of the Small and Long + Wide Greenhouses was almost constant despite variations in the amount of CO_2_ usage. In contrast, the inhomogeneity in the Long Greenhouse increased significantly as the CO_2_ usage increased. Their geometric features could cause this kind of difference, and it mainly depended on the area and aspect ratio (length: width).

Due to its smaller area, the Small Greenhouse exhibited a more uniform distribution of CO_2_ concentration, resulting in a smaller constant inhomogeneity. The Long + Wide Greenhouse had a similar aspect ratio but a larger area than the Small Greenhouse. As a result, it had a larger inhomogeneity but also at a constant. The main reason for this is that in the cultivation plane, CO_2_ diffusion can be considered a two-dimensional transport along with the greenhouse length and width directions. Therefore, greenhouses with similar aspect ratios have similar transport conditions, leading to constant inhomogeneities in both the Small and Long + Wide Greenhouses. However, the Long + Wide Greenhouse had a much larger area, which would cause a more serious concentration difference in the cultivation plane and result in a higher standard deviation in CO_2_ concentration. In contrast, the Long Greenhouse had an extremely higher aspect ratio than the other two greenhouses, which would limit the diffusion in the width direction. Therefore, CO_2_ diffusion can be seen as a one-dimensional transport along with greenhouse length. In addition, due to the dominance of CO_2_ gas generation over CO_2_ diffusion in terms of performance, CO_2_ gas tended to accumulate near the generator, particularly at the front. Consequently, as the amount of CO_2_ usage increased, the standard deviation of CO_2_ concentration expanded, reflecting the accumulation effect.

Notably, the ECE generally decreased as the CO_2_ usage increased. The two main reasons for the change in efficiency are as follows. First, a nonlinear relationship existed between leaf photosynthetic rate and CO_2_ concentration. When the CO_2_ concentration was initially low, increasing it could markedly enhance the photosynthetic rate, but as the concentration continued to rise, the effect gradually decreased^44^, resulting in reduced ECE. In response to this problem, maintaining the concentration in the cultivation area at a lower level seems to be more efficient under the current enrichment method. Second, the spatial distributions of CO_2_ concentration and photosynthetic rates within greenhouses are a potential reason. When the amount of usage was small, CO_2_ gas was mainly concentrated in the upper part of the greenhouses, so an increase in the concentration around the cultivation area (at 1.5 m height) was insignificant. As a result, the ECE became relatively low. As the amount of CO_2_ usage increased, more CO_2_ gas diffused from the upper part of the greenhouses to the cultivation area, and then efficiency was improved. Nevertheless, when further CO_2_ enrichment was continued, a significant portion of the CO_2_ gas was allocated to increasing the concentration in noncultivation spaces. This allocation is likely to have adverse effects on overall ECE.

### Eco-friendly strategy for CO_2_ enrichment in commercial greenhouses

By analysing the CO_2_, leaf photosynthetic rate distribution and ECE variations in different commercial greenhouses, the following problems were identified for the use of CO_2_ enrichment in commercial production:

The first is the uneven distribution of CO_2_ in the cultivation area. Using circulation fans to provide air movement seems to be an ideal method for solving this problem^45^. However, in this study, the cultivators did not use circulation fans in the greenhouses, possibly due to anticipated pest control difficulties. In addition, when using circulation fans, the airflow created by the circulation fans must be maintained at proper strength to avoid high wind speed damage to crops^46^.

Second, the existing enrichment method could not achieve a desirable increase in CO_2_ concentration in large-scale greenhouses. Therefore, burning more fuel to produce more CO_2_ from the generator seems the easiest way to solve this problem. However, simply increasing the CO_2_ supply from one generator may not be an ideal improvement measure because it would induce a serious uneven distribution of CO_2_, same with the Long Greenhouse. Therefore, increasing the number of CO_2_ generators and reasonably arranging their locations may be a better choice. In this case, more generators can ensure sufficient CO_2_ supply, and reducing the spacing between generators could effectively prevent evident CO_2_ unevenness inside the greenhouse.

Third, the limited distribution of CO_2_ at the height direction is another problem. Because the high temperature of CO_2_ gas caused this problem, lowering its temperature is the most direct solution. Zhang et al.^14^ proved the effectiveness of this measure in improving the distribution of CO_2_ in the height direction using CFD simulation.

Finally, through the quantitative analysis of ECE under different amounts of CO_2_ usage, setting the target CO_2_ concentration at a lower level of approximately 500 μmol mol^−1^ may be a better choice from an energy-efficiency perspective.

The above discussion of improvement measures was based on continually using the existing CO_2_ enrichment method. Various improvement measures may improve the application effect to a certain extent, but fundamental improvement is not guaranteed. Therefore, to make a qualitative change in the performance of CO_2_ enrichment in commercial production, the enrichment method needs to be fundamentally changed. A recently proposed new enrichment method (i.e. crop-local enrichment) may be an ideal solution^14,24,26,27^, as it enables CO_2_ gas transport to the cultivation area while directly releasing it into the crop canopy, thereby creating the highest CO_2_ concentration environment just around the strawberry canopy while ensuring good uniformity in the cultivation area.

## Conclusion

Spatial distribution of photosynthesis and CO_2_ within greenhouses under the CO_2_ enrichment was visualised using a CFD simulation–photosynthetic model. These distributions were characterised by the geometric features of greenhouses. Namely, the average value of CO_2_ concentration and photosynthetic rate decreased due to greenhouse scale from Small to the Large + Wide tests design issues, and the uniformity of their distribution deteriorated as the aspect ratio increased. In particular, for the Long Greenhouse, due to an imbalance of capacities of CO_2_ gas generation and transport, some areas had concentrations below and above the target concentration. However, most areas in the Long Greenhouse had a photosynthetic rate same as that of the Small Greenhouse, which can be attributed to the saturated relationship between photosynthetic rate and CO_2_ concentration. Furthermore, ECE peaked when the average CO_2_ concentration in the cultivation area was ~ 500 μmol mol^−1^ for Long and Long + Wide Greenhouses, and a subsequently continuous increase in the CO_2_ concentration would reduce efficiency. Considering the CO_2_ enrichment problems observed in commercial production, setting the target CO_2_ concentration to around 500 μmol mol^−1^ seems reasonable. Other measures, such as daytime running of circulation fans and crop-localised CO_2_ enrichment, could also be better choices, although they are yet to receive widespread applications in commercial greenhouses.

## Data Availability

The datasets used and/or analysed during the current study available from the corresponding author on reasonable request.
